# Examining the Use and Benefits of Low-/Mild-Gain Hearing Aids in Service Members with Normal Hearing Thresholds and Self-Reported Hearing Difficulties

**DOI:** 10.3390/healthcare12050578

**Published:** 2024-03-01

**Authors:** Alyssa J. Davidson, Gregory M. Ellis, Kimberly Jenkins, Melissa Kokx-Ryan, Douglas S. Brungart

**Affiliations:** 1 National Audiology and Speech Center, Walter Reed National Military Medical Center, Bethesda, MD 20889, USA; gellis@alakaina.com (G.M.E.); kimberly.a.jenkins33.civ@health.mil (K.J.); douglas.s.brungart.civ@health.mil (D.S.B.); 2 Alaka’ina Foundation Family of Companies Po’okela Solutions, Honolulu, HI 96814, USA; 3 National Intrepid Center of Excellence at Walter Reed National Military Medical Center, Bethesda, MD 20814, USA; melissa.j.kokx-ryan.civ@health.mil

**Keywords:** auditory processing, self-reported hearing difficulties, low-gain hearing aids, mild-gain hearing aids, management, hearing loss, correction of hearing impairment

## Abstract

Low- (or mild-) gain hearing aids (LGHAs) are increasingly considered for individuals with normal peripheral hearing but significant self-reported hearing difficulties (SHDs). This study assesses the benefits of LGHAs as a management option for individuals with normal hearing thresholds (NHTs) and SHDs, comparing LGHA use and benefit to individuals with non-significant hearing difficulties (NHDs) and those with peripheral hearing loss. Questionnaires addressing hearing aid usage, benefit, hearing difficulties, and tinnitus were administered to 186 individuals who self-identified as hearing aid users in a sample of 6652 service members who were receiving their annual hearing tests. Participants were divided into SHD and NHD groups based on the normative cutoff of the Tinnitus and Hearing Survey-Hearing Subscale (THS-H), and into hearing impairment (HI) and NHT based on their audiometric air-conduction thresholds. Individuals with SHDs and NHTs reported higher LGHA usage and benefit than individuals with NHDs and NHTs. Comparable use and benefit were noted between groups with SHDs regardless of peripheral hearing loss status. The findings support LGHAs as a suitable management option for individuals with NHTs and SHDs, as indicated by hearing aid use and benefit. Quantifying the level of perceived auditory processing deficits (i.e., SHDs), notably with the THS-H, enhances sensitivity in identifying those who may benefit the most from this treatment option.

## 1. Introduction

Auditory processing is a broad clinical term that refers to a patient’s ability to extract information from auditory signals detected at the auditory periphery. Auditory processing plays an important role in allowing listeners to extract features from sounds the listener wants to hear, like speech or music, as well as their ability to suppress interference from sounds they want to ignore, like interfering noise. Clinical definitions of auditory processing differ [1,2,3], but, in theory, impaired auditory processing could be a factor in all patients who experience hearing difficulties that are substantially worse than would be expected from their pure-tone audiometric thresholds. In practice, it is very difficult to develop tests that can distinguish between hearing difficulties resulting from impaired auditory processing and those resulting from peripheral hearing loss. Thus, the clinical diagnosis of auditory processing disorder (APD) has generally been limited to those patients who have normal hearing thresholds (NHTs) but exhibit worse than normal performance in suprathreshold listening tasks that involve sound localization, sound discrimination, temporal processing, pattern recognition, or the extraction of auditory signals from a noisy background. According to the American Academy of Audiology (AAA) Clinical Practice Guidelines [4] and as agreed upon by the European APD consensus [5], to diagnose APD, an individual has to perform at two standard deviations below the mean in at least one ear on two or more behavioral auditory tests. 

Historically, the clinical diagnosis and treatment of APD has been focused primarily on two groups: (1) children with abnormal hearing difficulties who were suspected of having congenital APD; and (2) adults with abnormal hearing difficulties who were suspected of suffering from acquired APD after experiencing some form of neurological trauma (e.g., stroke, Traumatic Brian Injury). However, in recent years, many research and clinicians have noted that some form of APD is probably also responsible for the problems reported by patients who seek help for their hearing difficulties in audiology clinics despite having NHTs. Some studies have estimated there could be as many as 26 million Americans who have NHTs but are suffering from significantly worse than expected hearing difficulties [6]. There is no clear consensus on how these patients should be classified and treated.

When considering APD in clinical treatment and management decisions, it is important to identify the goals of the audiological tests that will be administered in the clinic. Two potential approaches to evaluate APD include (a) performing a battery of tests to formally diagnose or (b) performing tests to evaluate a patient’s auditory ability in real-world listening environments. The tests used to formally diagnose an APD are designed to identify and classify specific abnormalities in the auditory nervous system. APD can result from various etiologies, including neurological factors (degenerative diseases, lesions, seizures, head trauma, and stroke) or developmental factors (e.g., delayed brain white matter maturation in children). There are some situations that require a formal APD diagnosis, such as diagnosis of a reimbursable disability in an adult or the development of an Individualized Educational Plan for a child. There may also be cases where the clinician is interested in identifying specific auditory processing areas where the patient is experiencing deficits. The American Speech-Language and Hearing Association provides guidelines for the formal diagnosis, treatment, and management of APD [7]. 

Many audiologists who have attempted to conduct comprehensive APD exams on individuals with significant self-reported hearing difficulties (SHDs) and NHTs have found that the results are inconsistent with the guidelines for identifying specific abnormalities in the auditory processing system, and that they frequently provide little insight into the best options for managing the symptoms reported by these patients. This has led audiologists to adopt a subset of auditory processing tests in their standard assessment batteries with the goal of evaluating individual abilities when hearing difficulties are reported. This allows clinicians to deduce if the patient has normal auditory processing abilities or not. 

Common objective auditory processing tests that assess temporal, binaural, and speech-in-noise processing abilities may reveal clinically abnormal performance in individuals with self-reported hearing difficulties and NHTs, but frequently they do not. Individuals with NHTs often exhibit a wide range of performance in these tests, and frequently individuals with self-reported hearing difficulties are found to perform at the low end of the normal range but not poorly enough to be considered clinically abnormal. This raises the question of whether the hearing difficulties patients report in the clinic can be considered “clinically significant” in the same way as an abnormal hearing score on a clinical hearing assessment. Verbal descriptions of hearing difficulties can be ambiguous, especially given that most audiologists may not have a lot of experience with the descriptions that patients without hearing difficulties would provide about the challenges of understanding speech in noisy or quiet environments. However, recent research suggests that questionnaires that evaluate patients’ self-reported hearing difficulties can be used to identify clinically significant problems when there is an appropriate normative sample of responses collected from a random sample of individuals with NHTs [8,9]. For example, a recent dissertation [10] reported using an abbreviated six-question version of the Speech, Spatial and Qualities of Hearing questionnaire (SSQ) [11] to evaluate blast-exposed service members (SMs) with NHTs for possible APD. The scores were compared to a fifth percentile cut-off value that was reported for a sample of 1943 SMs with NHTs and no history of blast exposure [12]. Individuals with SSQ scores below this threshold were considered to have SHDs, warranting further investigation and possible treatment for APD.

Currently, there is no clear consensus on how to treat individuals with NHTs who are found to have SHDs. Published studies have suggested several possible interventions for patients with abnormal auditory abilities, including compensatory strategies, counseling, and auditory training [13]. Several studies suggest that technologies that increase the signal-to-noise ratio (SNR), such as low- (or mild-) gain hearing aids (LGHAs), with or without the use of remote microphones, can provide significant benefits to those with SHDs and NHTs [14,15,16,17]. Anecdotal results from military audiologists have reported positive results from the use of LGHAs (i.e., self-reported satisfaction and use through data logging), and many military clinics have started routinely prescribing LGHAs to patients who present with SHDs and NHTs. This military population is one that has been reported anecdotally to have a relatively high proportion of individuals who have NHTs and SHDs [18,19,20]. The military population also differs from the civilian population in that SMs who are identified as hearing aid candidates may receive hearing aids free of charge. However, more evidence-based research is needed to evaluate the long-term effectiveness of LGHAs and to identify patients who are most likely to benefit from this intervention. 

The goal of the present study was to conduct a preliminary assessment of how LGHAs are being prescribed in the military population and estimate how much benefit they are providing for individuals with NHTs with and without SHDs. The assessment was conducted by surveying a large sample of individuals in a hearing conservation clinic in order to identify the relatively small proportion of individuals within the sample who had been prescribed hearing aids despite having NHTs. For comparison purposes, the sample also included more traditional hearing aid users with varying degrees of hearing impairment (HI). Self-reported hearing difficulties were assessed within both groups using the Tinnitus and Hearing Survey-Hearing Subscale (THS-H) [21]. The THS-H, a four-item validated questionnaire, instructs respondents to rate the level of difficulty experienced over the last week from 0 to 10 in various listening situations (i.e., in noisy or crowded places, on TV or in movies, people with soft voices, and group conversation). The cumulative sum of the responses quantifies self-reported hearing difficulties the individual is experiencing. Individuals were considered to have significant hearing difficulties (SHDs) if their total score on the THS-H exceeded 27, which placed in them the bottom fifth percentile of a sample of more than 15,000 participants with NHTs defined by ≤20 dB HL from 500 to 6000 Hz [21]. 

The primary research questions to be addressed in this retrospective study are (1) *How does use of and benefit from LGHAs in individuals with normal hearing thresholds and significant self-reported hearing difficulties (defined by their THS-H score) differ from those with non-significant self-reported hearing difficulties?* and (2) *How does use and benefit in those with normal hearing thresholds that were prescribed LGHAs differ from those who were prescribed hearing aids for peripheral hearing loss, regardless of degree of hearing difficulties?* It is hypothesized that (1) those with NHTs and SHDs will report more use and higher benefit than those with NHTs and non-significant hearing difficulties (NHDs); and (2) hearing aid use and benefit will be comparable across the groups of individuals with SHDs, regardless of peripheral hearing status (NHTs or HI).

## 2. Materials and Methods

Participants included in the study were SMs in the Department of Defense (DoD) hearing conservation program. Each year, SMs in this program undergo an automated air-conduction hearing threshold test spanning frequencies from 500 to 6000 Hz in both ears using the Defense Occupational and Environmental Health Readiness System-Hearing Conservation (DOEHRS-HC) system. Visual inspection of each ear is performed with an otoscope to rule out wax blockages and other obvious medical problems, but no bone conduction threshold testing, speech testing, or tympanometry is performed. Following the hearing test, SMs are given the opportunity to voluntarily participate in a research study. If they consent to be part of the study, the de-identified threshold results from the DOEHRS-HC test (500–6000 Hz) are integrated into the research component, which involves the completion of a set of questionnaires and listening tests requiring approximately 15 min of the participant’s time. The questionnaires encompass aspects such as age, sex, years of service, branch of service, language history, tinnitus complaints (e.g., THS-Tinnitus Subscale), hearing complaints (e.g., THS-H), hearing aid use, hearing aid benefit, and noise/blast exposure history. Some questionnaire items receive follow-up questions depending on the response (e.g., “Have you ever been prescribed hearing aids?” only receives follow-up if the SM responds “Yes”). Otherwise, all questionnaires are administered to all participants. It is important to note that researchers were not involved in clinical treatment or the SM’s decision to receive hearing aids. Additionally, there is no access to sensitive medical records or specific hearing aid records, (i.e., data logging) such as those stored in the NOAH system. The listening tests in the research portion commonly include assessments like an unaided speech-in-noise test and a binaural hearing test. 

There are both pros and cons to collecting data this way. Through this process, large-scale data are collected rapidly. Large datasets like this contribute to the understanding of various factors related to hearing health within the military population. Additionally, there is less probability of receiving biased questionnaire responses due to the anonymity of the research and separation from the SM’s provider. However, due to the way data are collected and the time constraints, some information that may be valuable are not available for analysis, such as the 8000 Hz air-conduction threshold, bone conduction thresholds, speech audiometry, objective data logging, or lengthy questionnaires.

The current study includes data collected from 6652 SMs who volunteered to participate in the study from 25 July 2022 to 28 November 2023. A *Hearing Aid Use and Benefit Questionnaire* was developed specifically for this study and was included in the research phase of data collection. The first question on the survey was a screening question for eligibility. If the SM answered “Yes,” they were included in the analysis and all following survey questions were asked. If the SM answered “No” or chose not to pursue, they were not included in the analysis and not administered any further questions on this survey. All participants were administered the THS-H regardless of hearing aid use. The following questions comprise the *Hearing Aid Use and Benefit Questionnaire* or the *Tinnitus and Hearing Survey-Hearing Subscale.*

*Hearing Aid Use and Benefit Questionnaire*

1. Have you ever been prescribed hearing aids?
  - Yes, and currently wear them full time.
  - Yes, and currently wear them intermittently or for select situations.
  - Yes, but no longer wear them.
  - No.
  - I’ve been recommended hearing aids but chose not to pursue.
2. Where did you receive your hearing aids?
  - At this DoD hospital or clinic.
  - At another DoD hospital or clinic.
  - At the Veteran’s Affairs (VA).
  - At a private audiology clinic.
3. What is/are the main reason(s) why you were prescribed hearing aids? [Select all that apply]
  - Tinnitus.
  - Difficulty hearing quiet sounds.
  - Difficulty understanding speech in noisy environments.
  - Other (with the option to type in a response).
4. For the following situations, rank the amount of benefit you receive from your hearing aids:
  - making it easier to tolerate tinnitus.
  - streaming music to the hearing aid.
  - streaming speech to the hearing aid from a phone or teleconference.
  - listening to speech in in-person meetings.
  - listening to speech in quiet environments.
  - listening to speech in moderately noisy environments with other talkers
  - listening to speech in loud vehicle or machinery noise.
  - listening to speech in very loud environments with other talkers (crowded happy hour).
  ■ Response options include:
    ○ No Benefit, Slight Benefit, Moderate Benefit, Substantial Benefit, or N/A
5. Approximately how many hours each day do you wear your hearing aids? [for each situation in Q4]
  ■ Response options include:
    - None or N/A, Less than 1 h, 1 to 4 h, 4 to 8 h, 8 to 16 h
  Questions and response options for this survey were developed based on internal suggestions from clinicians and researchers. Response options for hours of use each day were based on the International Outcome Inventory-Hearing Aids (IOI-HA) [22].

*Tinnitus and Hearing Survey-Hearing Subscale* [21]

1. Over the last week, I couldn’t understand what others were saying in noisy or crowded places.
  ■ Response options for each question are:
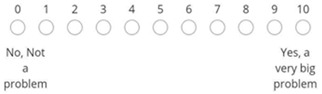
2. Over the last week, I couldn’t understand what people were saying on TV or in movies.
3. Over the last week, I couldn’t understand people with soft voices.
4. Over the last week, I couldn’t understand what was being said in group conversations.

A total of 186 participants were included based on answering “Yes” to the screening question. Participants were grouped based on peripheral hearing status and THS-H score. Based on the random selection process used to obtain the sample, data were collected for four groups of individuals: (1) those with NHTs (≤25 dB HL at 500, 1 kHz, 2 kHz, 3 kHz, 4 kHz, and 6 kHz in both ears) and SHDs (THS-H scores from 28 to 40); (2) those with hearing impairment (HI) and SHDs; (3) those with NHTs and NHDs (scores < 28 on the THS-H); and (4) those with HI and SHDs. The cutoff for hearing thresholds (≤25) was chosen for classification of NHTs based on in-house clinician report and classification for fitting LGHAs [10]. 

## 3. Analysis

Audiograms were analyzed using a Linear Mixed Effects Model (LMEM) to assess whether individuals with SHDs and individuals with NHDs had different audiometric thresholds. This is an important effect to control for, because if individuals with SHDs have worse thresholds than those with NHDs, any differences in reported hearing aid wear time or benefit could be due to elevated peripheral hearing thresholds in those with SHDs. Thresholds was the dependent variable. Independent variables included Hearing Impairment Status (NHT or HI), Hearing Difficulty (HD) Status (SHD or NHD), Ear (Better/Worse), and Frequency (500, 1 kHz, 2 kHz, 3 kHz, 4 kHz, and 6 kHz). Though the HI Status groups were defined based on their audiograms, the term was included in the model to account for variance related to those groups. Frequency was input to the model as a categorical variable to test for group differences at each of the six audiometric frequencies. The following fixed-effect terms were included in the model: Frequency, HD Status, HI Status, Ear, Frequency × HD Status, Frequency × HI Status, HD Status × HI Status, Ear × HD Status, Ear × HI Status, Frequency × HD Status × HI Status, and Ear × HD Status × HI Status. The random effects structure allowed intercepts to vary by subject, which was the maximal model that converged, following published guidelines [23]. The results of this analysis are reported in Section 4.1, Demographics.

Survey responses were analyzed using two separate Cumulative Link Mixed Models (CLMMs). CLMMs are appropriate for predicting ordinal data, like the survey responses used in the present study, and allow for the fitting of a random effects structure [24]. The data were analyzed using R version 4.2.1 and the *ordinal* package (version 2022.11-16) [25]. 

The first CLMM was performed with reported hourly hearing aid usage as the dependent variable. HI Status (NHT or HI), HD Status (SHD or NHD), and Situation (the eight listed above), were used as predictors. Because it was of theoretical interest to know whether members of each HI Status by HD Status group differed in their hearing aid use by Situation, the three-way interaction between HI Status, HD Status, and Situation was included in the model as well as all component two-way interactions. The random effects structure allowed intercepts to vary by subject. This was the maximum model that would converge, following the same guidelines as above [23]. The results of this analysis are reported in Section 4.3, Situational Hearing Aid Use.

The second CLMM was performed with reported benefit as the dependent variable. A CLMM was used instead of a linear model because it may not be the case that the difference between “No benefit” and “Slight benefit” is not the same as the difference between “Slight benefit” and “Moderate benefit.” The three-way interaction between HI Status (NHT or HI), HD Status (SHD or NHD), and Situation was included in the model with all component two-way interactions and main effects. The random effects structure allowed intercepts to vary by subject, the maximal model that would converge following the same guidelines as before [23]. The results of this analysis are reported in Section 4.4, Situational Hearing Aid Benefit.

## 4. Results

### 4.1. Demographics

**Figure 1:**
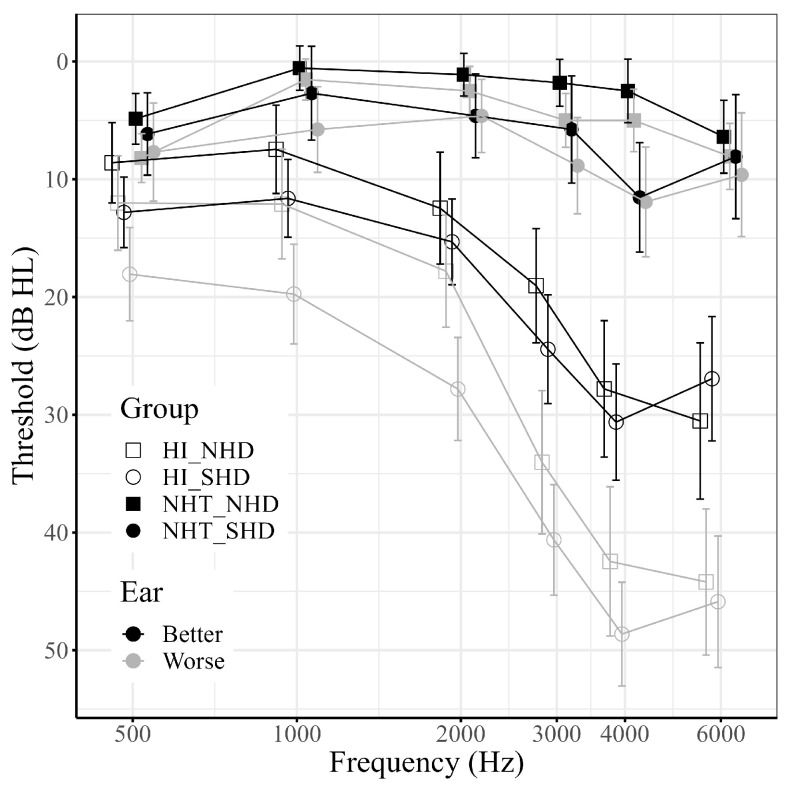
Mean hearing thresholds for better and worse ears across groups. Error bars represent 95% confidence intervals. Shape represents hearing difficulty group (squares: non-significant hearing difficulties, NHD; circles: significant hearing difficulties, SHD). Fill represents hearing status group (filled: normal hearing thresholds, NHT; open: hearing impaired thresholds, HI). Color represents ear (black: better ear; grey: worse ear). Note that within both the NHT and HI groups, SHD and NHD listeners have similar thresholds.

For the following analyses, participants were divided into four groups: NHT/SHD (n = 13); NHT/NHD (n = 36); HI/SHD (n = 80); and HI/NHD (n = 57). Demographics of the 186 participants separated by group can be found in Table 1. The average audiograms for better (B) and worse (W) ears for the groups can be found in Figure 1.

An LMEM was run in order to test for differences in audiometric thresholds between the SHD and the NHD groups. The full model was a better fit to the data than a constant model with only random intercepts by subject (χ^2^(27) = 1055.8, *p* < 0.001). The random effect also significantly improved the model (χ^2^(1) = 579.34, *p* < 0.001). Degrees of freedom were adjusted using Satterthwaite’s method. The following terms were significant: Ear × HI Status (*F*(1, 2045) = 40.56, *p* < 0.001), Frequency × HI Status (*F*(5, 2045) = 30.22, *p* < 0.001), Ear (*F*(1, 2045) = 79.59, *p* < 0.001), HI Status (*F*(1, 186) = 89.45, *p* < 0.001), and Frequency (*F*(5, 2330.9) = 62.0, *p* < 0.001). Neither the HI Status × HD Status interaction term (*F*(1, 186) = 0.09, *p* = 0.77) nor the HD Status main effect (*F*(1, 186) = 3.78, *p* = 0.053) were significant. No other terms were significant.

The main purpose of this LMEM was to assess whether the SHD and NHD groups differed in their thresholds. The results of the LMEM suggest that the SHD and NHD groups were matched on their peripheral hearing thresholds regardless of ear or frequency. The other effects are unsurprising: there is a significant difference between the better and worse ears of all groups and some frequencies have significantly different thresholds than others. Finally, HI Status groups had different thresholds, which stands to reason as these groups were defined based on their thresholds. Because these effects are not material to the main hypotheses of this paper, they were not explored further. 

### 4.2. Hearing Aid Current Wear and Reasons for Uptake

**Figure 2:**
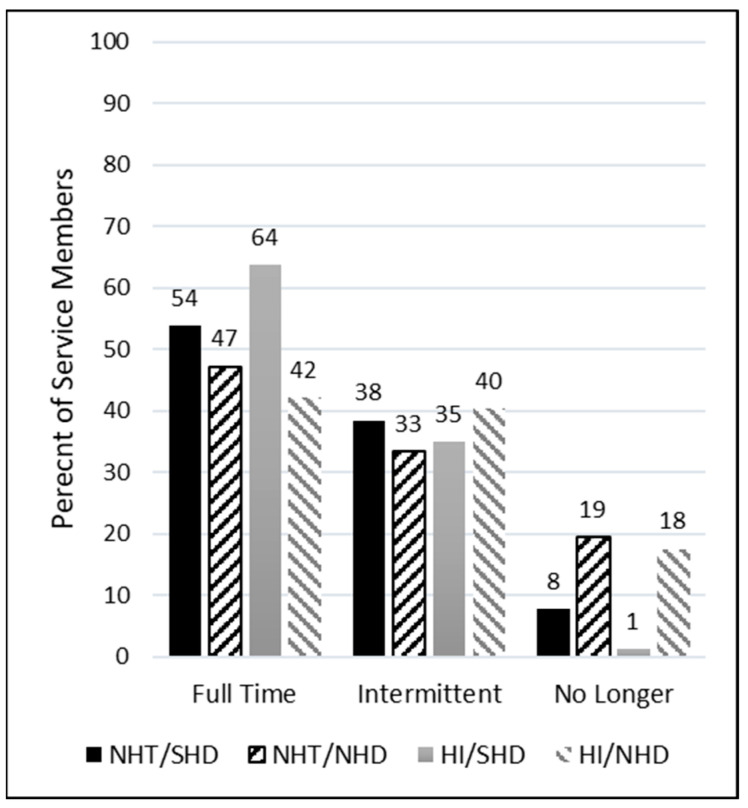
Percentage of service members by group who self-reported wearing their hearing aids full time, intermittently, or no longer.

Service members were asked how often they wear their current hearing aids with response options described previously. Figure 2 displays the distribution of self-reported responses for each group. All groups reported their highest proportion of use was full time. That is, most SMs used their hearing aids full time, regardless of peripheral hearing status. It is emphasized here that “full time” was not defined by a specific number of daily hours of use, but rather self-identified by the participant. However, the groups with non-significant self-reported hearing difficulties (NHT/NHD and HI/NHD) had substantially higher percentages of no longer wearing their devices compared to the groups with significant self-reported hearing difficulties (NHT/SHD and HI/SHD), regardless of hearing status. 

To assess the primary reasons why participants were prescribed hearing aids, three main categories were presented as options, along with an additional “Other” category (described previously). Participants had the flexibility to choose as many categories as they deemed applicable, resulting in varied representations across individuals and a total greater than the included number of participants. Note that 14 participants were removed from the dataset prior to analysis because their “Other” response indicated that they did not have hearing aids and chose the incorrect response. The remainder of the “Other” responses were either grouped into the respective categories (some wrote in “all three”) or left as “Other” when the response was left blank. 

**Figure 3:**
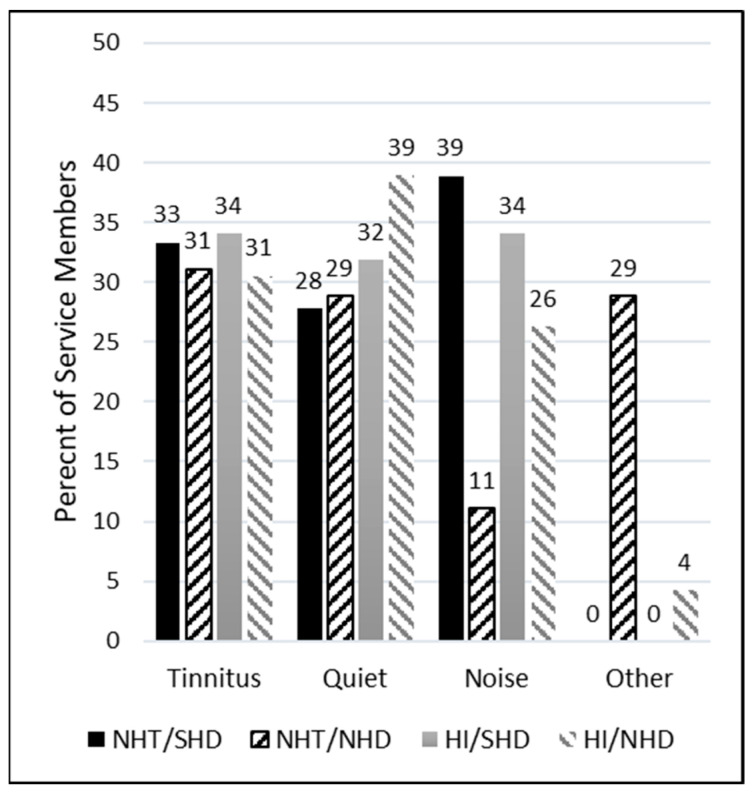
Percentage of service members by group who self-reported being prescribed hearing aids for tinnitus, understanding speech in quiet, understanding speech in noise, or for another unspecified reason.

As seen in the right column of Table 2, the predominant reasons selected for the prescription of hearing aids across all groups were *difficulty hearing quiet sounds* (n = 115) and *tinnitus* (n = 113). In the NHT groups, encompassing both SHD and NHD, *tinnitus* emerged as the primary reason hearing aids were prescribed (n = 20). Conversely, within the HI groups, the principal reason was *difficulty hearing quiet sounds* (n = 97). In the SHD groups, which included both NHT and HI individuals, *difficulty understanding speech in noisy environments* and *tinnitus* were the leading reasons (n = 71 and 70, respectively). In the NHD groups, the primary reason was *difficulty hearing quiet sounds* (n = 50). Figure 3 depicts the percentages of these observations across groups. Of note, among the NHT group with NHD, *difficulty understanding speech in noisy environments* was reported as a reason only 11% of the time. In contrast, the NHT group with SHD reported this difficulty more than three times as often (39% of the time). 

### 4.3. Situational Hearing Aid Use

**Figure 4:**
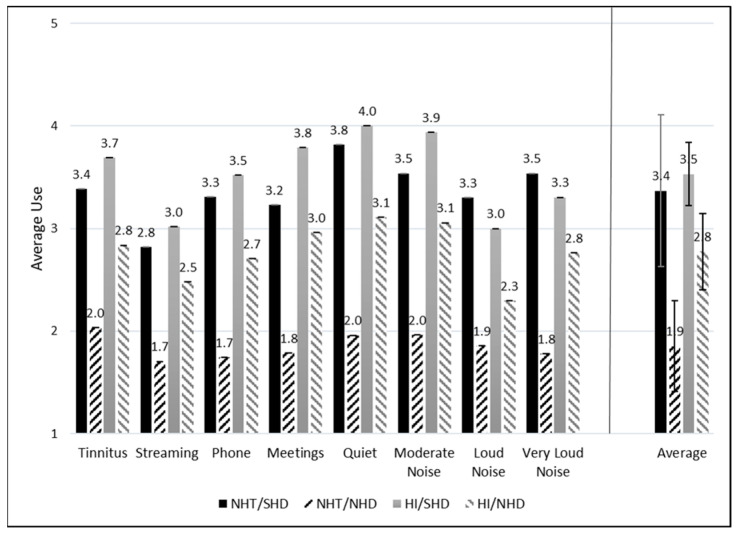
Average daily hearing aid usage across groups and listening situations. The average use across all situations for each group is displayed to the right of the vertical line. Standard error (SE) bars are depicted for 2 × SE.

**Figure 5:**
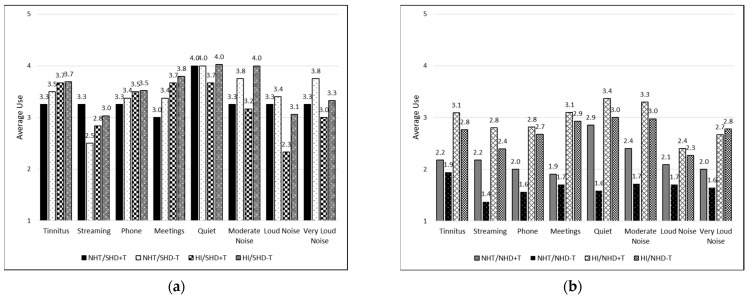
Average daily hearing aid usage across listening situations for those who reported tinnitus as the main and only reason for being prescribed hearing aids (+T) and those who did not report tinnitus as the only reason (−T). Groups are further divided: (**a**) participants with significant hearing difficulties (SHD) in the first panel; (**b**) participants with non-significant hearing difficulties (NHD) in the second panel.

Hearing aid use was categorized as described previously from none to 8 to 16 h of daily use. Each response was assigned a numeric value from 1 to 5 for the purpose of data visualization, with 1 representing “None or N/A”, 2 representing “Less than 1 h”, 3 representing “1 to 4 h”, 4 representing “4 to 8 h”, and 5 representing “8 to 16 h” (see Figure 4 and Figure 5). Eight situations were provided to each participant and they were able to decide if that situation applied to them or not. If the specific situation did not apply, N/A was selected by the participant and subsequently removed from the average and count. As such, not all situations had responses from every participant (refer to Table 3). 

Overall, *speech in moderately noisy environments with other talkers* was the situation participants indicated they used their hearing aids in most frequently (n = 175). *Speech in moderately noisy environments with other talkers* was also selected by the majority of participants in the NHT, SHD, and NHD groups (n = 41, 93, and 82, respectively). For the NHT group, *speech in in-person meetings* and *tolerating tinnitus* were also reported by the majority of participants (n = 41). *Speech in in-person meetings* was also highly reported in the SHD groups (n = 93). For the HI groups, *speech in quiet environments* was reported most frequently (n = 136). The situation with the lowest reported usage was *streaming music to the hearing aids* for all groups (n = 154). 

Figure 4 and Figure 5 depict the average amount of daily use for each situation separated by group. Higher numbers represent more self-reported hearing aid usage. For all situations, the SHD groups consistently report higher daily average usage as compared to the NHD groups. Those with NHT/SHD had more average usage across all listening situations compared to the NHT/NHD group. Similarly, those in the HI/SHD reported more average usage across situations than those in the HI/NHD group.

A CLMM was run to determine whether SMs reported different amounts of hearing aid use. A likelihood ratio test determined that the model was a significantly better fit than a constant model (χ^2^(31) = 254.74, *p* < 0.001). A second likelihood ratio test determined that the random effect of Subject also significantly improved the model (χ^2^(1) = 1087.5, *p* < 0.001). The coefficient for “Listening in quiet” was significantly different from the grand mean reported wear time (*β* = 1.16, *z* = 2.55, *p* = 0.01; OR = 3.19), indicating subjects were significantly more likely to report wearing their hearing aids longer in quiet than in other listening situations. There were also significant main effects of HD Status (*β* = 5.45, *z* = 4.39, *p* < 0.001; OR = 232.8) and HI Status (*β* = 3.60, *z* = 4.39, *p* < 0.001; OR = 36.6). The HD Status × HI Status interaction term was not significant (*β* = −2.5, *z* = −1.80, *p* = 0.07; OR = 0.08). No other interaction or main effect terms were significant.

When interpreting the results of this CLMM, the odds ratios are interpreted relative to the “None or N/A” category. Therefore, these results indicate three major points: (1) individuals were 3.19× as likely to wear their hearing aids in quiet than in other listening situations; (2) individuals with SHD were 232.8× more likely than individuals with NHD to report wearing their hearing aids; and (3) individuals with HI were 36.6× more likely than individuals with NHT to report wearing their hearing aids. The fact that the interaction term was not significant indicates that the HD Status and HI Status effects are additive; that is, individuals in the NHT/SHD category are the most likely of all four groups to report wearing their hearing aids.

Based on the high percentage of SMs who reported tinnitus as one of the potential multiple reasons for being prescribed hearing aids, it was of interest to evaluate a “tinnitus-only” group, that is, those who were prescribed hearing aids only for tinnitus. Within this study, 34% (15/63) of NHT participants and 6% (17/283) of HI participants reported tinnitus as the only reason for being prescribed hearing aids. However, all participants in this subgroup (n = 32) reported using their hearing aids in at least two other listening situations. 

For the tinnitus-only groups, average usage was compared between those who reported tinnitus as their only reason for being prescribed hearing aids (+T) and those who did not report tinnitus as their primary reason (−T) (i.e., tinnitus was indicated with another reason); see Figure 5a,b. This was further divided into those with NHT or HI and those with SHD or NHD. Figure 5a depicts those with SHD with consistently high usage for all situations except for streaming music to the hearing aids in the NHT/SHD−T and HI/SHD+T groups and speech in loud noise in the HI/SHD+T group. Conversely, in Figure 5b, which depicts those with NHD, usage is reported low across most listening situations.

### 4.4. Situational Hearing Aid Benefit

**Figure 6:**
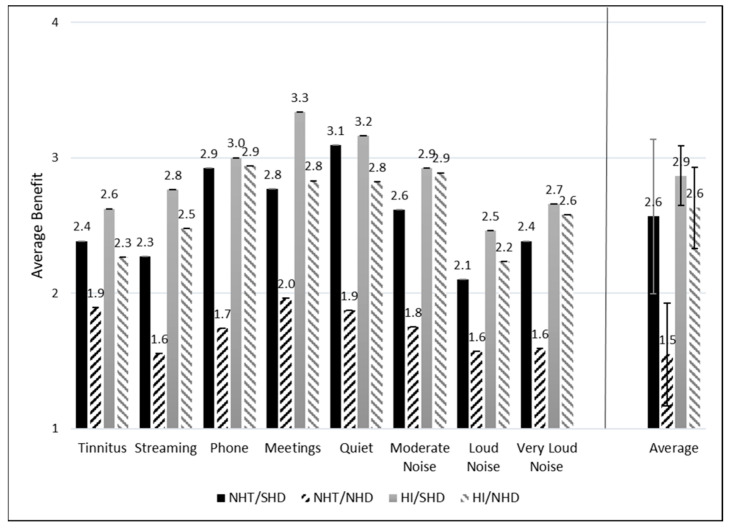
Average hearing aid benefit across groups and listening conditions. The average benefit across all situations for each group is displayed to the right of the vertical line. Standard error (SE) bars are depicted for 2 × SE.

**Figure 7:**
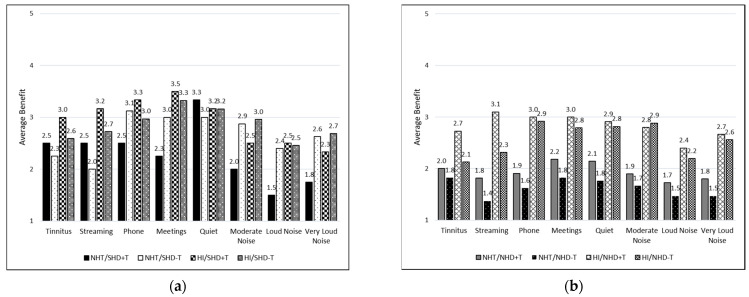
Average hearing aid benefit across listening situations for those who reported tinnitus as the main and only reason for being prescribed hearing aids (+T) and those who did not report tinnitus as the only reason (−T). Groups are further divided: (**a**) participants with significant hearing difficulties in the first panel (SHD); (**b**) participants with non-significant hearing difficulties (NHD) in the second panel.

Figure 6 and Figure 7 depict the average ratings of hearing aid benefit across groups for each listening situation. Each response was assigned a numeric value from 1 to 4 for the purpose of statistical analysis, with higher scores indicating more benefit. Higher benefit scores are reported for the NHT/SHD group compared to the NHT/NHD group when averaged across all listening situations. This difference is not observed when comparing the HI/SHD and HI/NHD groups. Figure 7 depicts the average benefit for those who may or may not have reported tinnitus as their primary reason for obtaining hearing aids. Specifically, Figure 7a represents those with SHD and Figure 7b represents those with NHD. Average benefit was overall higher for most listening situations for the SHD group compared to the NHD group, regardless of tinnitus being the primary reason for prescription. 

A second CLMM was run to determine whether SMs differed in their reported hearing aid benefit. Likelihood ratio tests indicated that the model was significantly better than a constant model (χ^2^(31) = 145.43, *p* < 0.001) and that the random effect also significantly improved the model (χ^2^(1) = 430.2, *p* < 0.001). The HI Status × APD Status interaction term of the model was significant (*β* = −2.40, *z* = −2.32, *p* = 0.02, OR = 0.09), as were both main effects for HI Status (*β* = 2.56, *z* = 3.99, *p* < 0.001, OR = 12.9) and APD Status (*β* = 3.02, *z* = 3.18, *p* = 0.001, OR = 20.5). No other main effect or interaction terms were significant predictors of hearing aid benefit.

When interpreting the results of this CLMM, the odds ratios are reported relative to no benefit. Therefore, the results of this CLMM indicate that individuals with SHD were 20.5× more likely than NHD individuals to report a benefit across all listening situations. Individuals with HI were 12.9× more likely than individuals with NHT to report a benefit. Importantly, the interaction term is significant and negative in this model, indicating that there is a moderated additive effect of being in the HI/SHD group, unlike the unmoderated additive effect in the previous CLMM. Here, individuals in the HI/SHD group are nearly 24× as likely as those in the NH/NHD group to report a benefit across all listening situations.

### 4.5. Global Hearing Aid Benefit 

**Figure 8:**
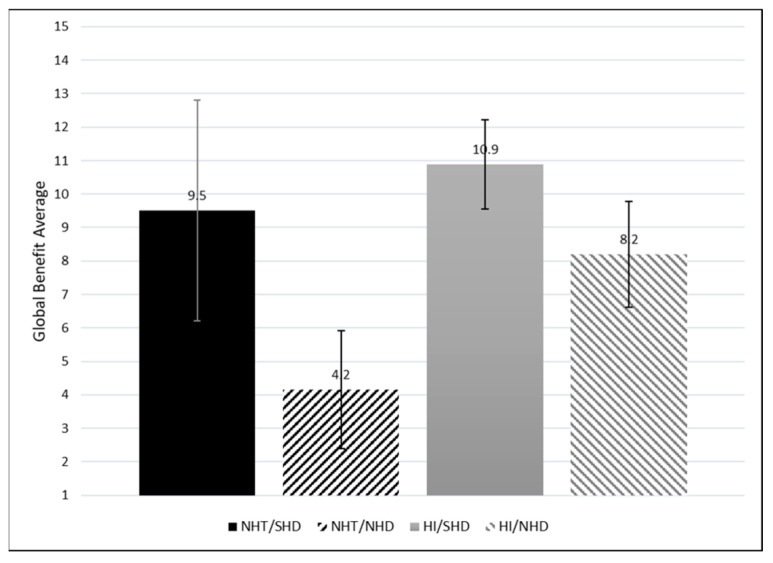
Average global hearing aid benefit across all listening situations for each group (Global = Avg Benefit × Avg Use). Standard error (SE) bars are depicted for 2 × SE.

To obtain a more comprehensive understanding of the benefits of hearing aids among these groups, a global score was calculated for benefit by adding benefit rating and use across all situations; see Figure 8. Weighting the global benefit by the reported use provides a total benefit score. A significant global benefit difference is observed between the NHT/SHD and NHT/NHD groups but not the HI/SHD and HI/NHD groups. This suggests that those with SHD have comparable global benefit, regardless of HI Status.

## 5. Discussion

Assessing auditory processing abilities or self-reported hearing difficulties constitutes an important component of the audiological assessment, particularly in the context of formulating management plans involving the potential inclusion of hearing aids. Utilizing standardized measures of self-reported hearing difficulties with published cutoffs can make it easier for clinicians to evaluate where a patient’s complaints fall relative to other individuals with similar hearing thresholds. In this study, the four-question THS-H survey and its published norms [21] were used to identify patients with SHDs or with NHDs. 

The study focused on two main questions: (1) *How does use of and benefit from LGHAs in individuals with normal hearing thresholds and significant self-reported hearing difficulties differ from those with non-significant self-reported hearing difficulties?* and (2) *How does use and benefit in those with normal hearing thresholds that were prescribed LGHAs differ from those who were prescribed hearing aids for peripheral hearing loss, regardless of hearing difficulties?*

The results of the study clearly support the use of hearing aids as a suitable management strategy for individuals with SHDs, irrespective of peripheral hearing status. Individuals with NHTs and SHDs had use and benefit reports that were comparable to individuals with HI and SHDs. A noteworthy revelation from this research is the higher incidence of discontinued hearing aid use among those with NHDs compared to their counterparts with SHDs, regardless of HI Status. Specifically, for Study Question 1, it was determined that participants with NHTs and SHDs reported significantly more hearing aid usage in all listening situations when compared to those with NHDs. For Study Question 2, results indicated that those with NHTs and SHDs had comparable reports of use and benefit to individuals with HI and SHDs. 

A comprehensive systematic review and meta-analysis, focusing on factors influencing hearing aid use or rejection, examined patterns of hearing aid usage for 12696 patients with and without hearing loss and a mean age of 72.7 years [26]. Their findings indicated that approximately 62% of individuals used their hearing aids, while 38% did not. In contrast, the prevalence of hearing aid use among the groups examined in the current study exceeded the systematic review estimate when considering full-time and intermittent use (defined by Q1 on the *Hearing Aid Use and Benefit Questionnaire* previously) combined (i.e., NHT/SHD: 92.3%; NHT/NHD: 80.6%; HI/SHD: 98.8%; HI/NHD: 82.5%). Within these delineated groups, a clear trend emerges, indicating that individuals with SHDs are more likely to persist in using their hearing aids compared to those with NHDs, regardless of peripheral hearing status. These compelling findings, coupled with variations in the age distribution and activities of daily living (e.g., retired vs actively working) of the studied populations, may offer insights into the differing observations regarding hearing aid usage and non-usage. 

Among these distinct groups, another interesting finding lies in the varied motivations for hearing aid adoption. Within the NHT cohort, the motivation for comprehending speech in noisy environments surfaced as a reason for seeking hearing aids 11% of the time for those with NHDs, while it notably increased to 39% for those with SHDs. A recent systematic review highlighted the significant factors contributing to hearing aid uptake [27]. Prominent among these influences were “self-reported hearing difficulties and beliefs” alongside “communication difficulties”. These findings align seamlessly with the current study, indicating that individuals reporting more challenges are seeking management solutions for communication in more demanding listening environments. This reinforces the notion that addressing specific communication challenges is a driving force behind the decision to pursue and continue to use hearing aid intervention, highlighting the nuanced motivations within different hearing profiles. 

To the extent that individuals with SHDs in the study appeared to obtain benefit from hearing aid use regardless of HI status, it is perhaps useful to see how many individuals in the sample with SHDs were currently using hearing aids. The data evaluated here were drawn from a total sample containing 6652 SMs, including 5278 with NHTs and 1374 with HI. The prevalence rates of SHDs based on the THS-H score was 9.1% (480/5278) in the NHT group and 24.4% (356/1374) in the HI group. Of these SMs with hearing difficulties, only 16% of the individuals in the HI/SHD group (57/356) were currently using hearing aids, and only 2.7% of the NHT/SHD group (13/480) were currently using hearing aids. While it is true that many factors go into the determination of hearing aid candidacy, it seems at least possible that there are a number of SMs with SHDs who might benefit from the use of hearing aids, including many who have NHTs. Systematic administration of a hearing difficulty survey like the THS-H might make it possible to identify NHT individuals who might benefit from a referral to audiology to be evaluated for possible hearing aid candidacy. 

The Tinnitus Subscale of the Tinnitus and Hearing Survey (THS-T) was also administered to participants who reported experiencing tinnitus. Within the current study groups, the full THS-T results were available for 10/13 (NHT/SHD), 7/36 (NHT/NHD), 47/80 (HI/SHD), and 33/57 (HI/NHD) SMs. Clinical guidelines for the THS [28] suggest that only those individuals with a higher THS-T score than a THS-H should receive treatment that is primarily focused on tinnitus rather than hearing loss. Within this study, 18 participants fell in this category. Of these, five participants reported that tinnitus was the only reason they were prescribed hearing aids, six reported tinnitus as one of the reasons but not the only reason, and seven did not indicate tinnitus at all. Curiously, the proportion of individuals with THS-T scores greater than THS-H stores who reported tinnitus was their primarily reason for getting hearing aids (27%) was very similar to the overall percentage of individuals with NHT who said the tinnitus was the primary reason for getting hearing aids (31%). This would seem to suggest that the THS-T > THS-H relationship was not a good predictor of whether individuals are prescribed hearing aids for tinnitus or for hearing loss. However, one must acknowledge the possibility that individuals who received some relief from tinnitus as a result of hearing aids may have reported lower THS-T scores in this survey than they did at the time the audiologist initially prescribed the hearing aids. 

This brings us to one of the limitations of the approach used in this study, which is that there is some ambiguity about whether the participants in this study were answering the THS-H (and THS-T) questions with regard to the difficulties they were experiencing without their hearing aids or whether they responded with regard to the difficulties they experienced when they were wearing their hearing aids. There is no question that there was a trend for individuals who reported greater difficulties to also report greater benefit, which suggests either that these individuals were reporting based on their difficulties without their hearing aids or that their hearing difficulties were enormous when the hearing aids were not used. Either of these possible alternatives would still lead to the conclusion that individuals who report SHDs on self-reported questionnaires are likely to be good candidates for hearing aids. However, the quantitative relationship between THS-H score and hearing aid benefit might be warped if the responses are related to the difficulties experienced when the hearing aids were worn. 

Another possible limitation of the study is that the use of a sample of military personnel may limit the extent to which these results can be generalized to the civilian population. Military members may experience unique noise exposures, like explosive blasts, that are very uncommon in any civilian population, and they are likely to experience exposures like gunfire and heavy equipment noise at a higher rate than their civilian counterparts [29]. Without further research on an equivalent civilian population, it will be difficult to know if these results hold for civilians, particularly with regard to the benefit of prescribing LGHAs to NHT individuals with SHDs. It is worth noting, however, that SMs and Veterans represent about 10% of the adult population in the US and probably a much higher proportion of the individuals who are candidates for hearing aids, so these results are still likely to be directly applicable to a large number of patients in military and civilian audiology clinics. 

Other limitations of the study include the necessity of patient self-report to infer the reason why they were recommended for hearing aids and for reporting hearing aid use. There is no way to tell whether the individuals were subjected to formal APD testing, or how they might have performed on any speech-in-noise testing that was conducted during their clinical appointments. The results would be more specific to APD if at least one behavioral auditory test was evaluated on all study participants (e.g., evaluating speech in noise/babble, dichotic digits, and temporal resolution abilities). This limitation is evident in our attempts to identify individuals who may have been prescribed hearing aids in order to address problems with tinnitus rather than speech-in-noise problems. Very few patients reported they were prescribed hearing aids only for tinnitus, but patients are known to sometimes confound problems with tinnitus and hearing difficulties, and it is possible that a significant proportion of the NHT/NHD group were prescribed hearing aids solely as a remedy for tinnitus. Although it would be preferable to have objective data logging to support the self-reported daily hours of hearing aid use, these data were not available. In future studies, this limitation will be taken into consideration with the addition of an audiologist-focused questionnaire to input hours from hearing aid software. 

A final comment should be made on the *Hearing Aid Use and Benefit Questionnaire*, which was developed for use in this study but has not yet been validated against other hearing aid benefit surveys or against quantitative hearing aid use data based on data logging. Although other instruments were considered, none were identified that addressed the particular use and benefit scenarios we were interested in for LGHA uses. The survey was created based on clinician and hearing aid researcher inputs and was designed to be a comprehensive survey to address use and benefit in specific situations that is not available in other published questionnaires such as the IOI-HA. A future initiative will be to validate this questionnaire against the IOI-HA in addition to objective data logging. Additionally, future research will evaluate the benefits of hearing aids in those with APD, defined by the clinical guidelines [4], combined with self-reported hearing difficulties to shed further light on possible management decisions. 

## 6. Conclusions

Anecdotal reports from military audiologists suggest that, as a result of the increased military activity that occurred after 2001, there has been an increase in the number of SMs with NHTs but reports of SHDs commensurate with individuals with some degree of hearing loss. These difficulties appear to be particularly prevalent in individuals with a history of blast exposure [12]. In response to this problem, many of these audiologists, particularly in the DoD, have started to prescribe LGHAs to these patients. However, this approach has not been universally accepted. Within the audiology community, there has been debate about the appropriateness of prescribing hearing aids to individuals who do not have elevated peripheral hearing thresholds. There are also no guidelines for when to prescribe LGHAs, which was a driving question this study wanted to begin to address. With regard to this question, the results of this study are unambiguous. Individuals who had NHTs but reported difficulties severe enough to place them in the SHD category used their hearing aids as frequently and received roughly the same amount of benefit from their hearing aids as those who had peripheral hearing loss. They also used their hearing aids in similar situations, and obtained similar amounts of benefit from their hearing aids in those situations. This included listening in quiet environments, where one might expect individuals with elevated thresholds to obtain a much larger benefit due to enhanced audibility. More research is still needed to fully understand why these individuals receive benefits from LGHAs across a wide range of listening environments, but, in terms of clinical outcomes, it would be hard to argue that individuals in the NHT/SHD category are not obtaining at least a significant perceived benefit from the use of these devices. It should also be noted that, within the sample of 5278 NHT SMs in the survey, 480 had hearing complaints severe enough to qualify for the SHD category but only 13 (roughly 2.7%) had been prescribed hearing aids. This seems to indicate the possibility that there are many SMs with NHTs in the active-duty population who could benefit from the use of hearing aids, and that the THS-H is a viable option to indicate the need for referral However, more data are needed to explore this possibility in more detail.

The hearing aid benefits for individuals with NHT who had THS-H scores to put them in the NHD category are somewhat more ambiguous. Many of these individuals may have been prescribed their hearing aids primarily for tinnitus, and individuals in the NHD/NSD+T group did report a greater benefit for tinnitus than for any other listening situation. However, their overall hearing aid usage benefit was much lower than for any of the other groups tested in this study. This may be because they are no longer experiencing difficulty, and thus not wearing their device at the time of the research due to successful rehabilitation. It is interesting that this group was much more likely than the other groups to have received their hearing aids from a private practice rather than a DoD or VA clinic. This appears to contradict the notion that the DoD and VA are outliers in terms of their propensity to subscribe hearing aids to patients with NHTs. 

Perhaps the greatest implication of this study is that it highlights the benefits of using a standardized measure of self-reported hearing difficulty, like the THS-H, to assess hearing aid candidacy, particularly in patients with NHTs. Individuals in the SHD group, who had scores greater than 27 points on the THS-H, were much less likely to report that they were no longer wearing their hearing aids (Figure 2). Within the NHT group, they also wore the hearing aids much more often and received much greater overall benefit. With more data, it might be possible to develop a better way to predict hearing aid benefit with the THS-H than to simply use a single cutoff value to identify individuals who are likely to receive a greater benefit from hearing aids. However, the results from this study do suggest that the inclusion of a simple, standardized self-reported hearing difficulty survey like the THS-H should be collected along with the audiogram in cases where individuals in a large population of listeners are being screened for their hearing aid candidacy. 

## Figures and Tables

**Table 1:** Demographics of participants.

Characteristic	NHT/SHD (n = 13)	NHT/NHD (n = 36)	HI/SHD (n = 80)	HI/NHD (n = 57)
Gender				
Men	12 (92%)	33 (92%)	72 (90%)	55 (96%)
Women	1 (8%)	3 (8%)	8 (10%)	2 (4%)
Age Groups				
18–30	6 (46%)	25 (69%)	23 (29%)	20 (35%)
31–40	5 (39%)	10 (28%)	33 (41%)	26 (46%)
41–50	2 (15%)	1 (3%)	21 (26%)	10 (18%)
51–60	0 (0%)	0 (0%)	3 (4%)	1 (2%)
Years of Service				
1–5	3 (23%)	20 (57%)	7 (9%)	13 (23%)
6–10	4 (31%)	8 (23%)	16 (20%)	12 (21%)
11–20	4 (31%)	7 (20%)	35 (44%)	22 (39%)
21+	2 (15%)	0 (0%)	22 (28%)	10 (18%)
Received Devices				
DoD	11 (84%)	27 (75%)	74 (93%)	53 (93%)
VA	1 (8%)	1 (3%)	2 (2%)	1 (2%)
Private Practice	1 (8%)	8 (22%)	4 (5%)	3 (5%)

SHD = significant hearing difficulties; HI = hearing impaired; NHD = non-significant hearing difficulties; NHT = normal hearing thresholds.

**Table 2:** Counts of the main reasons why participants were prescribed hearing aids. Multiple categories could be selected.

Reason	NHT/SHD (n = 13)	NHT/NHD (n = 36)	HI/SHD (n = 80)	HI/NHD (n = 57)	Total NHT/HI	Total SHD/NHD	Total All
Tinnitus	6 (33%)	14 (31%)	64 (34%)	29 (31%)	20 (32%)/93 (33%)	70 (34%)/43 (31%)	113 (33%)
Difficulty hearing quiet sounds	5 (28%)	13 (29%)	60 (32%)	37 (39%)	18 (29%)/97 (34%)	65 (32%)/50 (36%)	115 (33%)
Difficulty undestanding speech in noisy environments	7 (39%)	5 (11%)	64 (34%)	25 (26%)	12 (19%)/89 (31%)	71 (34%)/30 (21%)	101 (29%)
Other	0 (0%)	13 (29%)	0 (0%)	4 (4%)	13 (21%)/4 (1%)	0 (0%)/17 (12%)	17 (5%)
Totals	18	45	188	95	63/283	206/140	346

**Table 3:** Counts of the situations in which participants reported using their hearing aids.

Situation	NHT/SHD (n = 13)	NHT/NHD (n = 36)	HI/SHD (n = 80)	HI/NHD (n = 57)	Total NHT/HI	Total SHD/NHD	Total All
Making it easier to tolerate tinnitus	13 (13%)	28 (13%)	77 (13%)	49 (12%)	41 (13%)/117 (12%)	90 (13%)/68 (11%)	158 (12%)
Streaming music to the hearing aid	11 (11%)	27 (12%)	68 (11%)	48 (12%)	38 (12%)/116 (11%)	79 (11%)/75 (12%)	154 (12%)
Streaming speech to the hearing aid from a phone or teleconference	13 (13%)	27 (12%)	75 (12%)	48 (12%)	40 (13%)/123 (12%)	88 (12%)/75 (12%)	163 (12%)
Speech in in-person meetings	13 (13%)	28 (13%)	80 (13%)	53 (13%)	41 (13%)/133 (13%)	93 (13%)/81 (13%)	174 (13%)
Speech in quiet environments	11 (11%)	24 (11%)	80 (13%)	56 (14%)	35 (11%)/136 (13%)	91 (13%)/80 (13%)	171 (13%)
Speech in moderately noisy environments with other talkers	13 (13%)	28 (13%)	80 (13%)	54 (13%)	41 (13%)/134 (13%)	93 (13%)/82 (13%)	175 (13%)
Speech in loud vehicle or machinery noise	10 (10%)	28 (13%)	76 (12%)	51 (12%)	38 (12%)/127 (13%)	86 (12%)/79 (13%)	165 (12%)
Speech in very loud environments with other talkers	13 (13%)	27 (12%)	79 (13%)	50 (12%)	40 (13%)/129 (13%)	92 (13%)/77 (12%)	169 (13%)
Totals	97	217	615	409	314/1015	712/617	1329

## Data Availability

The data presented in this study may be available on request from the corresponding author.
